# Functional and phosphoproteomic analysis of β-adrenergic receptor signaling at excitatory synapses in the CA1 region of the ventral hippocampus

**DOI:** 10.1038/s41598-023-34401-7

**Published:** 2023-05-09

**Authors:** Shekib A. Jami, Brent J. Wilkinson, Ryan Guglietta, Nicolas Hartel, Walter E. Babiec, Nicholas A. Graham, Marcelo P. Coba, Thomas J. O’Dell

**Affiliations:** 1 Molecular, Cellular, and Integrative Physiology Interdepartmental PhD Program, University of California, Los Angeles, Los Angeles, CA USA; 2 Zilkha Neurogenetic Institute, Los Angeles, CA USA; 3 Interdepartmental PhD Program for Neuroscience, University of California, Los Angeles, Los Angeles, CA USA; 4 Mork Family Department of Chemical Engineering and Materials Science, University of Southern California, Los Angeles, CA USA; 5 Undergraduate Interdepartmental Program for Neuroscience, University of California, Los Angeles, Los Angeles, CA USA; 6 Department of Psychiatry and Behavioral Sciences, Keck School of Medicine, University of Southern California, Los Angeles, CA USA; 7 Department of Physiology and Neuroscience, Keck School of Medicine, University of Southern California, Los Angeles, CA USA; 8 Integrative Center for Learning and Memory, Brain Research Institute, University of California, Los Angeles, Los Angeles, CA USA; 9 Department of Physiology, David Geffen School of Medicine, University of California, Los Angeles, Los Angeles, CA USA

**Keywords:** Neuroscience, Cellular neuroscience, Synaptic plasticity, Synaptic transmission

## Abstract

Activation of β-adrenergic receptors (β-ARs) not only enhances learning and memory but also facilitates the induction of long-term potentiation (LTP), a form of synaptic plasticity involved in memory formation. To identify the mechanisms underlying β-AR-dependent forms of LTP we examined the effects of the β-AR agonist isoproterenol on LTP induction at excitatory synapses onto CA1 pyramidal cells in the ventral hippocampus. LTP induction at these synapses is inhibited by activation of SK-type K^+^ channels, suggesting that β-AR activation might facilitate LTP induction by inhibiting SK channels. However, although the SK channel blocker apamin enhanced LTP induction, it did not fully mimic the effects of isoproterenol. We therefore searched for potential alternative mechanisms using liquid chromatography-tandem mass spectrometry to determine how β-AR activation regulates phosphorylation of postsynaptic density (PSD) proteins. Strikingly, β-AR activation regulated hundreds of phosphorylation sites in PSD proteins that have diverse roles in dendritic spine structure and function. Moreover, within the core scaffold machinery of the PSD, β-AR activation increased phosphorylation at several sites previously shown to be phosphorylated after LTP induction. Together, our results suggest that β-AR activation recruits a diverse set of signaling pathways that likely act in a concerted fashion to regulate LTP induction.

## Introduction

Noradrenergic neurons in the locus coeruleus project widely throughout the central nervous system and, through the release of the modulatory neurotransmitter norepinephrine (NE), regulate crucial aspects of brain function, including attention, emotional arousal, sensory processing, and memory formation^1^. The noradrenergic regulation of memory formation is especially striking, as essentially every aspect of learning and memory, including acquisition, consolidation, retrieval, re-consolidation, and extinction is potently modulated by NE^1–3^. Although NE can modulate neuronal activity via activation of both α and β-adrenergic receptor (β-AR) subtypes, noradrenergic regulation of learning and memory importantly (although not exclusively) involves β-ARs^3–5^. For example, β-AR antagonists inhibit the acquisition and/or consolidation of fear conditioning^6,7^, spatial learning^8^, and other forms of associative learning^9–14^. Inhibition of β-ARs also disrupts memory retrieval^15,16^, re-consolidation^17–19^, and extinction^20,21^. β-adrenergic receptor activation also enhances the induction and maintenance of activity-dependent forms of synaptic plasticity thought to be involved in memory formation, such as NMDA receptor (NMDAR)-dependent long-term potentiation (LTP)^4,5^. At many excitatory synapses, NE appears to act in strictly modulatory fashion. For example, β-AR activation can increase the potentiation induced by patterns of synaptic activity that are near threshold for LTP induction^22–28^ and facilitate the induction of long-lasting, protein synthesis-dependent forms of LTP by patterns of synaptic stimulation that normally induce shorter-lasting LTP^29,30^. However, at other synapses β-AR activation does not serve a strictly modulatory role but instead acts as an essential factor (in addition to coincident pre and postsynaptic activity) required for LTP induction. For example, β-AR activation is required for the induction of spike-timing-dependent LTP at excitatory synapses onto pyramidal cells in visual cortex^31–33^ and medial prefrontal cortex^33^. Moreover, the β-AR antagonist propranolol blocks high-frequency stimulation-induced LTP at excitatory synapses onto dentate gyrus granule cells^34,35^ and inhibits LTP induction in the CA1 region of the ventral hippocampus^36^.

The modulation of LTP induction by β-AR activation is thought to importantly involve activation of protein kinase A (PKA) and the extracellular signal-regulated kinases ERK1/2 followed by phosphorylation-dependent changes in the activity of postsynaptic glutamate receptors, voltage-activated ion channels, and signaling pathways controlling protein synthesis and gene expression^4,5^. Whether these same mechanisms also underlie the role of β-AR signaling at synapses where activation of these receptors is required for LTP induction is less well understood. Notably, in contrast to CA1 pyramidal cells in the dorsal hippocampus, the induction of LTP at Schaffer collateral (SC) synapses onto pyramidal cells in the ventral hippocampus is highly dependent on β-AR activation^36^. Thus, to investigate the signaling mechanisms underlying plasticity at synapses where activation of β-ARs has an especially prominent role in LTP induction we examined LTP induction and β-AR signaling at SC synapses in the ventral hippocampus. Importantly, at some synapses, including SC synapses in the ventral hippocampus^37^, LTP induction is suppressed by activation of SK-type calcium-activated potassium channels^38,39^. Because activation of β-ARs and PKA inhibits SK channel activity at excitatory synapses^39,40^, we initially investigated whether SK channels are a key synaptic target where β-adrenergic receptor signaling can act to facilitate LTP induction. Consistent with previous results^36^, we find that theta-frequency stimulation induced-LTP at SC synapses in the ventral hippocampus requires β-AR activation. However, the selective SK channel blocker apamin only partially mimicked the ability of β-AR activation to enable LTP induction at these synapses. Thus, to identify potential alternative mechanisms underlying β-AR-dependent forms of LTP we used liquid chromatography-tandem mass spectrometry (LC/MS–MS) to measure changes in phosphorylation of postsynaptic density (PSD) proteins induced by the β-AR selective agonist isoproterenol (ISO) in isolated CA1 regions obtained from ventral hippocampal slices. This analysis indicates that β-AR activation has a surprisingly widespread effect on PSD proteins, bi-directionally regulating phosphorylation at several hundred sites found in a wide variety of PSD proteins. Increases in phosphorylation triggered by β-AR activation are predicted to be mediated by multiple families of protein kinases and occur at sites within the core scaffold machinery of the PSD that partially overlap with sites phosphorylated following the induction of LTP^39^. Our results thus suggest that the ability of β-AR activation to regulate protein phosphorylation of the PSD protein interaction network (PIN) in a way that partially recapitulates changes that occur during LTP induction may importantly contribute to LTP induction in the ventral hippocampus.

## Results

### β-AR activation enables TPS-induced LTP at SC fiber synapses onto CA1 pyramidal cells in the ventral hippocampus

**Figure 1:**
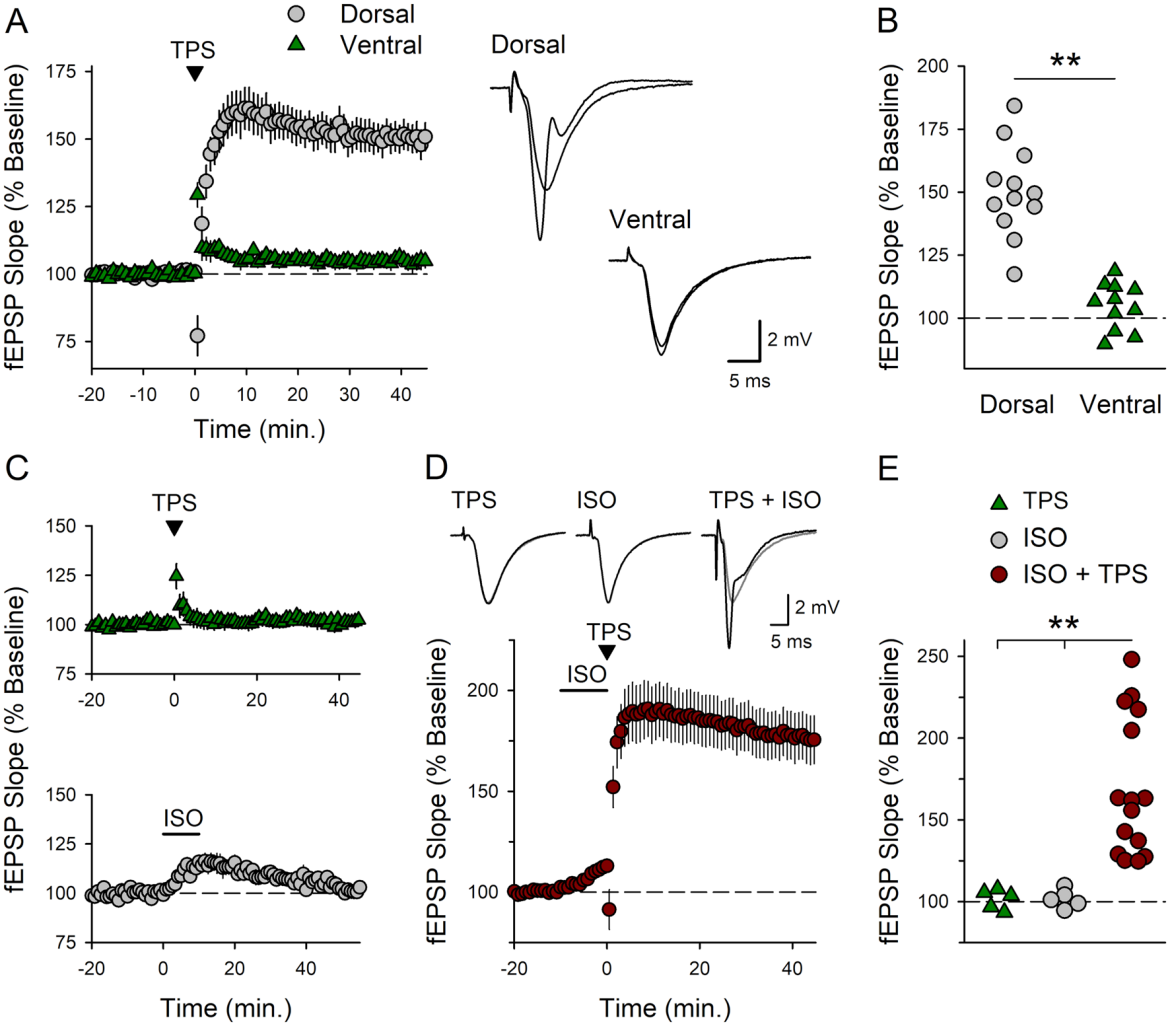
LTP induction in the ventral hippocampal CA1 region requires β-adrenergic receptor activation. (**A**) A 30-s-long train of TPS (delivered at time = 0) induced robust LTP in dorsal hippocampal slices (fEPSPs were potentiated to 150 ± 5% of baseline, n = 12) but had no lasting effect on synaptic strength in ventral hippocampal slices (fEPSPs were 105 ± 3% of baseline, n = 11). Traces show superimposed fEPSPs recorded during baseline and 45 min post-TPS in dorsal and ventral slices. (**B**) Scatter plot shows fEPSP slopes 45 min post-TPS in all experiments (t(21) = 7.447, **p = 2.55 × 10^–7^). (**C**) Synaptic transmission in ventral hippocampal slices was not persistently altered by either TPS alone (30 s duration, top) or bath application of 1.0 µM ISO alone (bottom). Field EPSPs were 101 ± 2% of baseline 45 min after TPS (n = 5) and 101 ± 3% of baseline 45 min after ISO application (n = 5). (**D**) TPS delivered at the end of a 10-min bath application of ISO (indicated by the bar) induced robust LTP (fEPSPs were potentiated to 176 ± 12% of baseline, n = 16). Traces show superimposed fEPSPs recorded during baseline and 45 min post-TPS alone, ISO alone, or TPS delivered in the presence of ISO. (**E**) Scatter plot shows fEPSP slopes 45 min post-TPS or ISO application in all experiments. **p < 0.005, one-way ANOVA with post hoc Bonferroni t-tests, F_(2,23)_ = 11.396, p < 0.001.

Patterns of synaptic stimulation that induce robust LTP at SC synapses onto CA1 pyramidal cells in the dorsal hippocampus typically produce relatively modest or no potentiation at these synapses in the ventral hippocampus^37,43,44^ (however, see^45^). Consistent with this, activation of SC synapses with a 30-s-long train of theta pulse stimulation (TPS) induced LTP at SC synapses in dorsal hippocampal slices maintained in-vitro but had no lasting effect on synaptic transmission at SC synapses in slices from the ventral hippocampus (Fig. 1A, B). To determine whether β-AR activation is required for TPS-induced LTP in the ventral CA1 region we next compared how synaptic strength is modified by either TPS alone, bath application of ISO alone (1.0 µM for 10 min), or TPS delivered in the presence of ISO. As expected, 30 s of TPS alone had no lasting effect on synaptic strength (Fig. 1C). Moreover, although field excitatory postsynaptic potentials (fEPSPs) were modestly enhanced in the presence of ISO, synaptic strength returned to baseline levels following ISO washout (Fig. 1C). However, consistent with the notion that TPS-induced LTP is dependent on β-AR activation, TPS delivered in the presence of ISO induced robust LTP (Fig. 1D, E).

**Figure 2:**
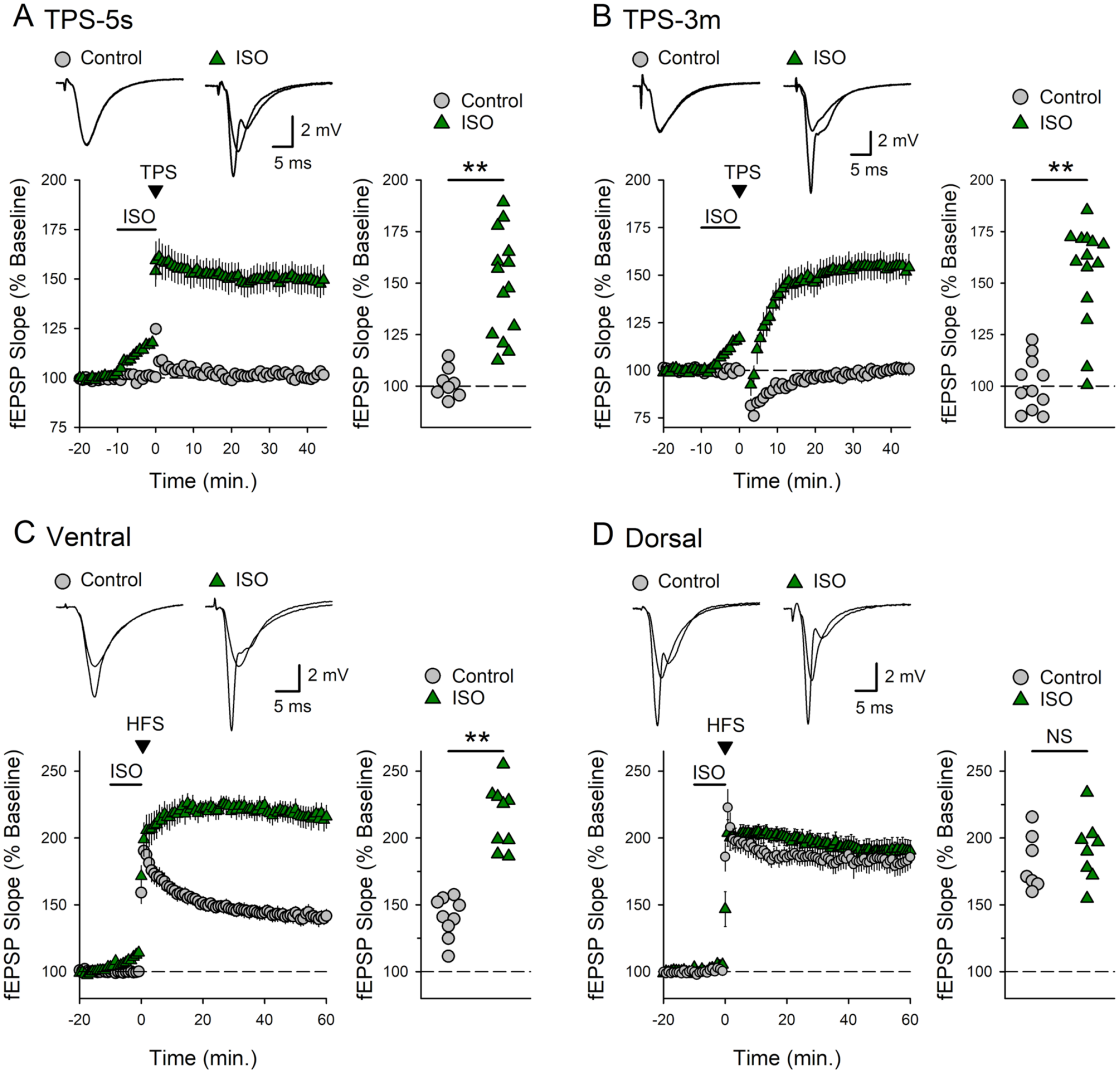
β-adrenergic receptor enables TPS and HFS-induced LTP in the ventral hippocampus. (**A**) A brief train of TPS (5 s) had no lasting effect on synaptic strength in control experiments (fEPSPs were 101 ± 2% of baseline 45 min post-TPS, n = 10) but induced LTP when delivered in the presence 1.0 µM ISO (fEPSPs potentiated to 149 ± 7% of baseline, n = 14, t(20) = 5.881, **p = 6.43 × 10^–6^, compared to control). (**B**) β-adrenergic receptor activation enables the induction of LTP by a longer train of TPS (3 min). fEPSPs were 101 ± 4% of baseline in control experiments (n = 11) and 155 ± 7% of baseline in experiments were TPS was delivered in the presence of ISO (n = 14, t(23) = 6.502, **p = 1.24 × 10^–6^, compared to control). (**C**) In ventral hippocampal slices fEPSPs were potentiated to 141 ± 5% of baseline in control experiments (n = 9) and 216 ± 8% of baseline when HFS was delivered in the presence of ISO (n = 9). (**D**) In dorsal hippocampal slices fEPSPs were 182 ± 8% of baseline 60 min post-HFS in control experiments (n = 7) and 191 ± 8% of baseline when HFS was delivered in the presence of ISO (n = 8). Scatter plots show fEPSP slopes 45 min post-TPS (**A** and **B**) or 60 min post-HFS (**C** and **D**) in all experiments. For results shown in (**C**) and (**D**), statistical significance was determined using a two-way ANOVA and post hoc Bonferroni t-tests. There was a significant effect of region (F_(1,29)_ = 32.764, p < 0.001) and a significant region × ISO interaction (F_(1,29)_ = 20.052, p < 0.001). Although ISO did not enhance LTP in dorsal hippocampal slices (NS, p = 0.406), ISO enhanced LTP induction in slices from the ventral hippocampus (**p < 0.001). In control experiments, HFS-induced LTP was significantly smaller in ventral compared to dorsal hippocampal slices (p < 0.001). Traces in (**A**–**D**) show superimposed fEPSPs recorded during baseline and 45 min post-TPS (**A**, **B**) or 60 min post-HFS (**C**, **D**).

In the dorsal CA1 region, the induction of LTP by TPS exhibits a pronounced inverted-U shaped dependence on TPS train duration, with trains lasting 15–30 s being optimal for LTP induction^46,47^. Thus, to determine whether β-AR-dependent LTP in the ventral hippocampus exhibits a similar dependence on TPS train duration we next examined how SC synapses in the ventral CA1 region are modified by both brief (5 s) and prolonged (3 min) trains of TPS delivered in the presence and absence of ISO. Both of these patterns of TPS had no lasting effect on synaptic strength when delivered in the absence of ISO but induced significant LTP when delivered in the presence of ISO (Fig. 2A, B). This indicates that β-AR activation has an essential role in the induction of LTP by strikingly different patterns of synaptic stimulation in the ventral hippocampus. Indeed, β-AR activation also significantly facilitated the modest potentiation induced by high-frequency SC fiber stimulation in the ventral hippocampus (Fig. 2C). In contrast, HFS alone induced a robust potentiation of synaptic transmission in dorsal hippocampal slices that was not enhanced when HFS was delivered in the presence of ISO (Fig. 2D). Along with previous findings^36^, these results indicate β-AR activation has an essential role in the induction of LTP in the CA1 region of the ventral hippocampus.

**Figure 3:**
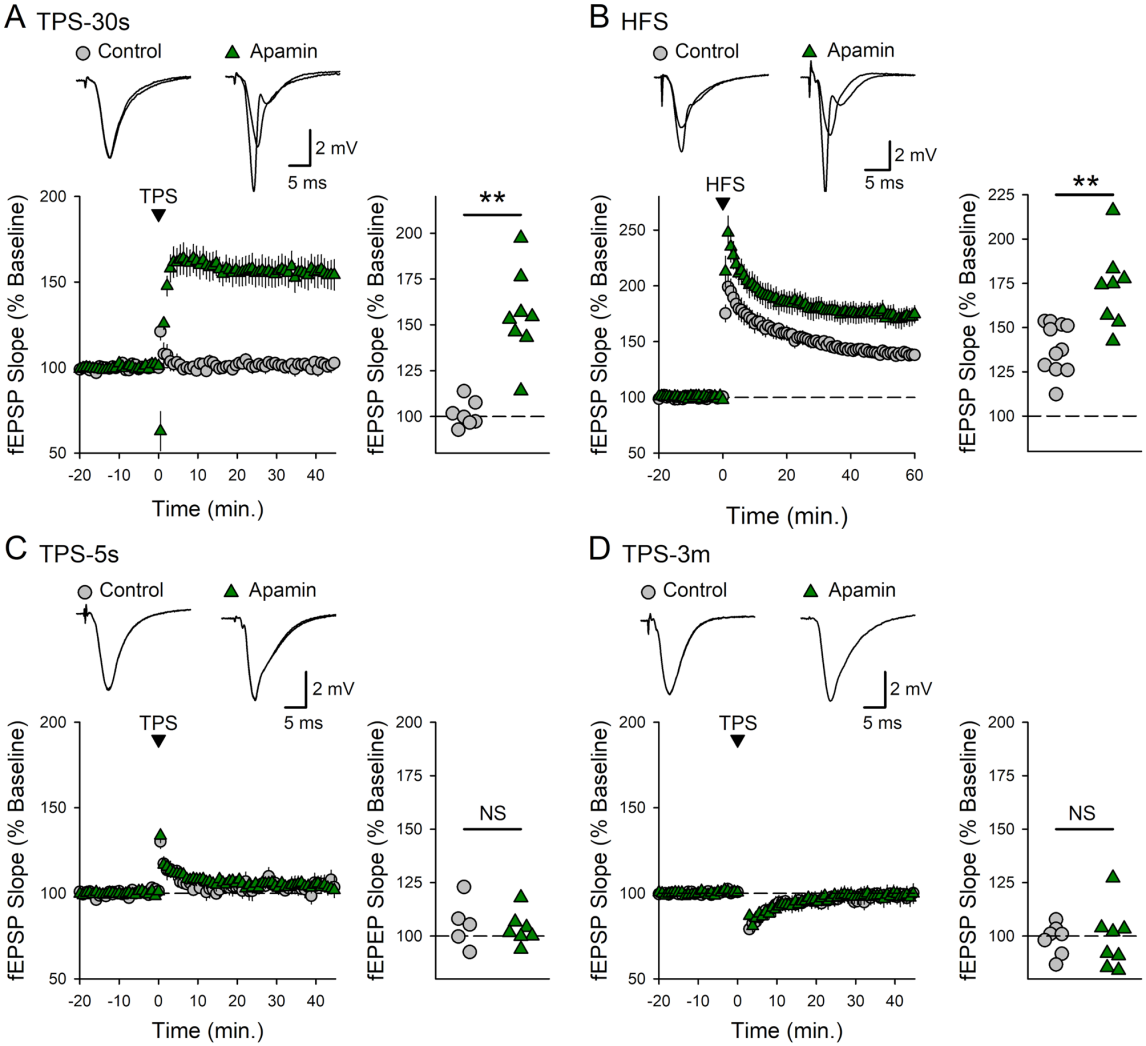
Blocking SK channels only partially mimics the ability of β-adrenergic receptor activation to enable LTP induction in ventral hippocampal slices. (**A**) A 30 s-long train of TPS had no persistent effect on synaptic strength in control experiments (45 min post-TPS fEPSPS were 101 ± 3% of baseline, n = 7) but induced LTP in slices continuously bathed in ACSF containing SK channel blocker apamin (100 nM) (fEPSPs potentiated to 155 ± 9% of baseline, n = 8, t(13) = 5.622, **p = 8.31 × 10^–5^ compared to control). (**B**) Apamin enhances HFS-induced LTP. Field EPSPs were potentiated to 139 ± 4% of baseline in control experiments (n = 11) and 172 ± 8% of baseline in experiments where SK channels were blocked with apamin (n = 8, t(17) = 4.014, *p = 9.0 × 10^–4^ compared to control). (**C** and **D**) Apamin does not enable the induction of LTP by either brief (**C**) or long trains of TPS (**D**). Following a 5 s train of TPS, fEPSPs were 106 ± 5% of baseline in control experiments (n = 5) and 103 ± 3% of baseline in apamin-treated slices (n = 7, t(10) = 0.424, p = 0.68 compared to control). fEPSPs were 98 ± 3% of baseline 45 min after 3 min of TPS in control experiments (n = 7) and 99 ± 5% of baseline in slices bathed in ACSF containing apamin (n = 8, t(13) = 0.0217, p = 0.983 compared to control). Scatter plots show fEPSP slopes 45 min post-TPS or 60 min post-HFS in all experiments.

Activation of SK-type potassium channels strongly opposes NMDA receptor activation and prevents TPS-induced LTP at SC synapses in the ventral hippocampus^37^. Thus, downregulation of synaptic SK channel activity following β-adrenergic receptor activation^40^ may provide a key mechanism whereby NE could enable LTP induction at these synapses. If so, pharmacological inhibition of SK channels should be sufficient to enable LTP in the ventral CA1 region. Consistent with this notion, bath application of the selective SK channel inhibitor apamin (100 nM) not only enabled the induction of LTP by 30 s of TPS (Fig. 3A) but also enhanced HFS-induced LTP at SC synapses in the ventral hippocampus (Fig. 3B). However, unlike β-AR activation, inhibition of SK channels did not enable the induction of LTP by brief (5 s.) or prolonged (3 min.) trains of TPS (Fig. 3C, D). Thus, although SK channel inhibition may contribute to the induction of β-adrenergic receptor-dependent LTP, modulation of SK channel activity alone cannot account for the crucial role of β-AR activation in the induction of LTP at SC synapses in the ventral hippocampus.

### Regulation of PSD protein phosphorylation following β-AR activation

**Figure 4:**
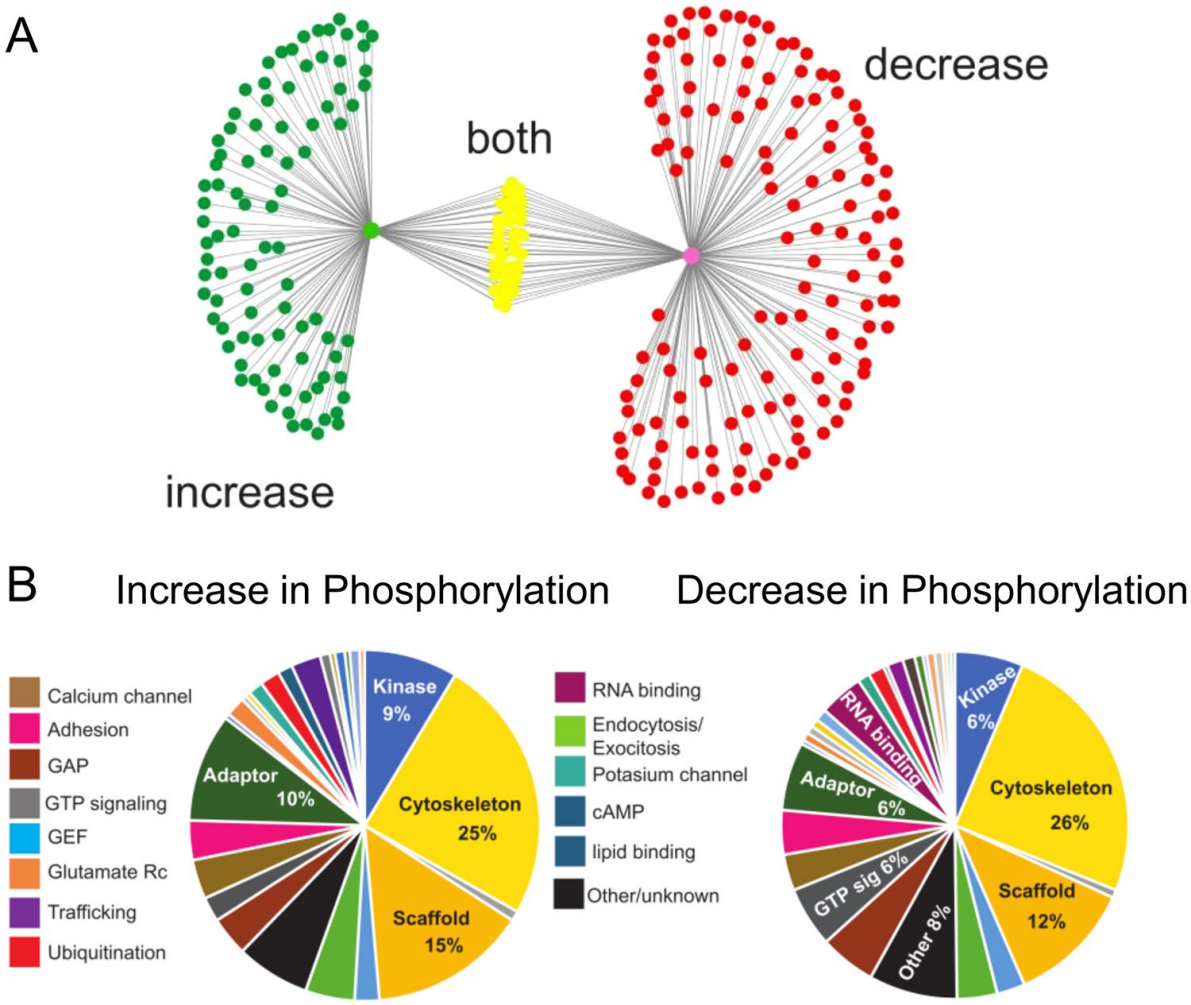
Functional classification of PSD proteins with phosphorylation sites modified by β-AR activation. (**A**) Distribution of proteins exhibiting increases (93), decreases (138), or bidirectional (39) changes in phosphorylation following β-AR activation. (**B**) Pie charts show functional classification for phosphorylated (left) or dephosphorylated proteins following β-AR activation.

To gain insights into alternative signaling mechanisms that might contribute to β-AR-dependent forms of LTP, we used LC–MS/MS to identify changes in phosphorylation of PSD proteins induced by the β-AR agonist ISO in CA1 mini-slices obtained from the ventral hippocampus. We identified 3321 phosphorylation sites in PSD proteins of which 561(16.9%) were regulated following β-AR activation (Supplementary Table 1). Consistent with conventional view that β-AR activation regulates LTP induction by phosphorylating synaptic proteins, we observed increased phosphorylation at 262 sites in 132 proteins (Supplementary Tables 2, 3). However, β-AR activation also triggered dephosphorylation at a similar number of sites (299 phosphorylation sites in 177 proteins) (Supplementary Tables 4, 5). Of the 270 PSD proteins regulated by β-AR activation, 39 contained sites that were phosphorylated and other sites that were dephosphorylated. (Fig. 4A). Although β-AR activation modulated phosphorylation sites in PSD proteins with a wide variety of functions, more than 50% of the sites upregulated by ISO where found in cytoskeleton proteins (25%), protein scaffolds (15%), adaptor proteins (10%), and protein kinases (9%) (Fig. 4B). A similar pattern was seen for sites dephosphorylated by β-AR activation (Fig. 4B). β-AR activation regulated phosphorylation sites in proteins involved in diverse forms of signaling within the PSD, including protein phosphatases, Ca^2+^/calmodulin signaling, and regulators of small GTPases (Supplementary Table 6). Consistent with the notion that phosphorylation-dependent modulation of ligand and voltage-gated ion channels is responsible for the effects of β-AR activation on LTP induction, ISO triggered bidirectional changes in phosphorylation of glutamate receptor subunits as well as α and β subunits of both K^+^ and Ca^2+^ channels (Supplementary Table 6). Although we did not detect phosphorylated SK channels, β-AR did regulate three phosphorylation sites in the α subunit of large-conductance, BK-type calcium-activated K^+^ channels. Consistent with previous findings indicating that phosphorylation of AMPAR GluA1 subunits at S845 has a crucial role in the β-AR modulation of LTP induction^26,29,46^, β-AR activation increased GluA1 phosphorylation at this site (Supplementary Table 6). Moreover, β-AR activation also increased phosphorylation at sites in the cytoplasmic C-terminus of AMPAR GluA2 (S901) and GluA3 (S885) subunits. Phosphorylation sites in the cytoplasmic C-terminus of NMDAR GluN2A and 2B subunits were also regulated by β-AR activation. Notably, β-AR activation extensively regulated phosphorylation of α and β subunits of voltage-dependent Ca^2+^ channels, increasing phosphorylation of three sites in L-type channel (Ca_v_1) β2 and β4 subunits, decreasing phosphorylation of three sites in the α subunits of R-type channels (Ca_v_2.3), and bidirectionally regulating multiple sites (increased phosphorylation at three sites and decreased phosphorylation at four sites) in α subunits of P/Q-type channels (Ca_v_2.1).

### PSD protein kinases regulated by β-AR activation

**Figure 5:**
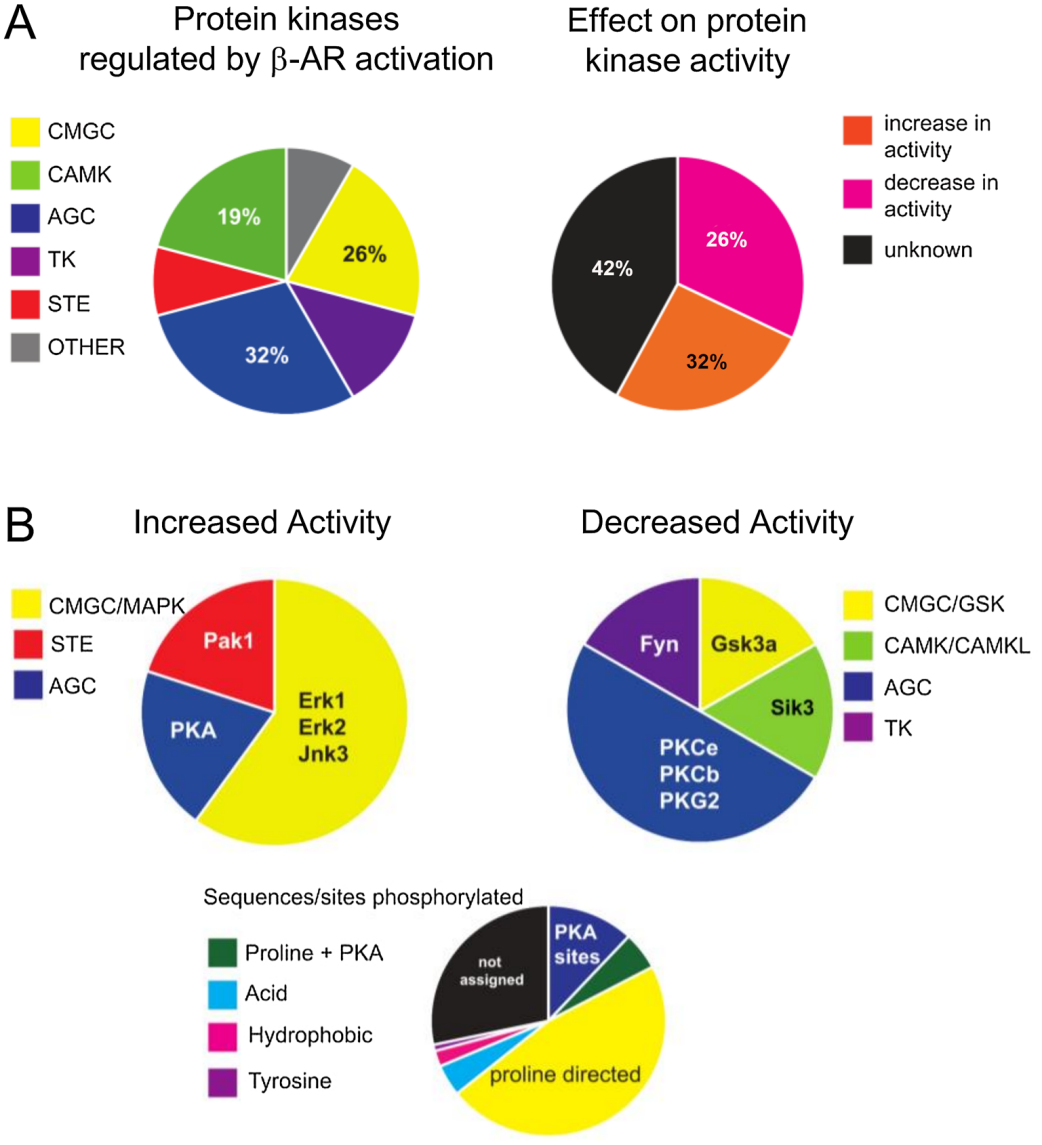
Protein kinase families and protein kinases regulated by activation of β-ARs. (**A**) Left: distribution of regulated kinases within each group: CMGC (named after CDK, MAPK, GSK3 and CLK group of protein kinases), STE (named after homologues sterile yeast kinases), AGC (named after Protein kinase A, G and C), TK (tyrosine kinases) CAMK (Calcium/calmodulin protein kinases). Right: predicted effect of β-AR activation on protein kinase activity. (**B**) Top: charts show protein kinases and protein kinase families with increases (left) and decreased (right) in protein kinase activity induced by β-AR. Bottom: predicted kinases for phosphorylation sites regulated by β-AR activation based on flanking amino acid sequence analysis.

To identify the β-AR-regulated protein kinases involved in phosphorylation of PSD proteins we first examined how β-AR activation regulates phosphorylation sites within PSD protein kinases. Strikingly, although just two protein kinases (PKA and ERK1/2) have been implicated in the β-AR modulation of LTP induction^4^, β-AR activation regulated phosphorylation sites in 24 PSD protein kinases (Supplementary Table 7). These protein kinases were predominantly distributed among three groups, AGC (32% sites), CMGC (26% sites) and CAMK group (19% sites) (Fig. 5A). Because protein kinase activity is frequently regulated by phosphorylation in the kinase catalytic domain, we used both our MS analysis and western blotting (Supplementary Table 7) to identify changes in kinase catalytic domain phosphorylation associated with changes in catalytic activity. From the total of 31 phosphorylation sites in protein kinases that were regulated by β-AR activation, 32% of the changes in phosphorylation were found at sites correlated with an increase in protein kinase activity and 26% occurred at sites associated with a decrease in activity (Fig. 5A, Supplementary Table 7). Our analysis indicates that three major protein kinase groups contribute to increased protein kinase activity following β-AR activation: the CMGC kinases Erk1, Erk2, and Jnk3; the STE kinase Pak1; and, as expected, the AGC kinase PKA (Fig. 5B). Conversely, β-AR activation induced changes in phosphorylation associated with decreased protein kinase activity in AGC kinases PKCe, PKCb, PKG, and Akt1, the CMGC kinase GSK3α, the tyrosine kinase Fyn, and CAMK kinase Sik3 (Fig. 5B).

To gain additional insight into the kinases regulating phosphorylation of PSD proteins following β-AR activation, we, first, searched for previously identified protein kinase-substrate pairs reported in the literature using the PhosphositePlus database (https://www.phosphosite.org). However, we found that only a very low percentage (10%) of quantitated p-sites have kinase-substrate pairs that have been previously assigned. Thus, we next used the Eukaryotic Linear Motif ELM resource^49^ and Networkin^50^ to predict protein kinases for each individual phosphorylation site, followed by manual curation of each phosphorylation site, to identify protein kinase consensus sequences surrounding the phosphorylation sites upregulated by β-AR activation (Supplementary Table 8). We found that the amino acid sequence motifs flanking phosphorylated sites corresponded to the families of kinases upregulated by activation of β-ARs (Fig. 5B). The largest consensus sequence motifs corresponded to proline directed kinases (CMCG group) and PKA consensus sequences, with 47% of phosphorylation sites occurring within the proline directed kinases motif (X-X-X-S/T-P-X-X-X) and 12% occurring with the PKA consensus sequence (R-X-X-S/T-ϕ-X-X, where ϕ is a hydrophobic amino acid) and an additional 5% occurring within a combined proline directed/PKA consensus motif (MAPK/PKA: R-X-X-S/T-P-X-X). Other, less abundant kinase consensus sequence motifs detected in PSD proteins phosphorylated in response to β-AR activation included phospho-tyrosine sites,consensus acid (Ck1/Ck2) and hydrophobic motifs, as well as basophilic motifs that are typically preferred by protein kinases form the AGC group, such as PKA (Fig. 5B, Supplementary Table 8). Together, our analysis of phosphorylation site consensus motifs corresponds well with the identification of activated kinases determined by protein kinase regulatory site phosphorylation. Moreover, these results are consistent previous results showing that activation of PKA and ERK1/2 has an essential role in the β-AR-mediated facilitation of LTP induction^4^.

### β-AR activation regulates phosphorylation of core PSD scaffolding complex proteins

Previously, we found that the induction of LTP by high-frequency synaptic stimulation prominently regulates, and primarily increases, phosphorylation of scaffolding proteins in the PSD^41^. Thus, to examine whether β-AR activation also regulates PSD core scaffolding complex proteins involved in LTP, we examined the effects of ISO on phosphorylation of the five main scaffolding complex proteins: the disk large proteins (DLGs), members of the SHANK family of proteins (Shank1-3), and the disk large-associated guanylate-associated (DLGAP) protein family (DLGAP1-4), along with the associated proteins SynGAP1 and Lrrc7 (Densin-180) (Supplementary Table 9). β-AR activation had little effect on phosphorylation of DLG proteins, increasing phosphorylation at just two sites in Dlg4 (PSD95) and decreasing phosphorylation at two sites in PSD95 and one site in Dlg2 (PSD93). A larger number of regulated phosphorylation sites were found in DLGAPs (increases in phosphorylation at five total sites (two sites in Dlgap1, one in Dlgap3, and two sites in Dlgap4; and dephosphorylation at one site in Dlgap2). In contrast, the Shank family of proteins exhibited a relatively large number of phosphorylation sites regulated by β-AR activation, with ISO inducing increases at 10 sites (four sites in SHANK1, two in SHANK2 and four in SHANK3) and dephosphorylation at 18 sites (three in SHANK1, eight sites in SHANK2, and seven sites in SHANK3). Phosphorylation of the scaffolding complex associated proteins Lrrc7 and SynGAP1, which are regulated following HFS-induced LTP^39^, was also bidirectionally regulated by β-AR activation (four sites up-regulated and one site dephosphorylated in Lrrc7 and three sites upregulated and three sites dephosphorylated in SynGAP1). This suggests that β-adrenergic receptor activation might be regulating protein phosphorylation within a subset of phosphorylation sites previously found to be regulated by the induction of LTP, and specifically being modulated by CamKIIα activation.

### β-AR activation regulation of protein phosphorylation within the PSD protein interaction network

**Figure 6:**
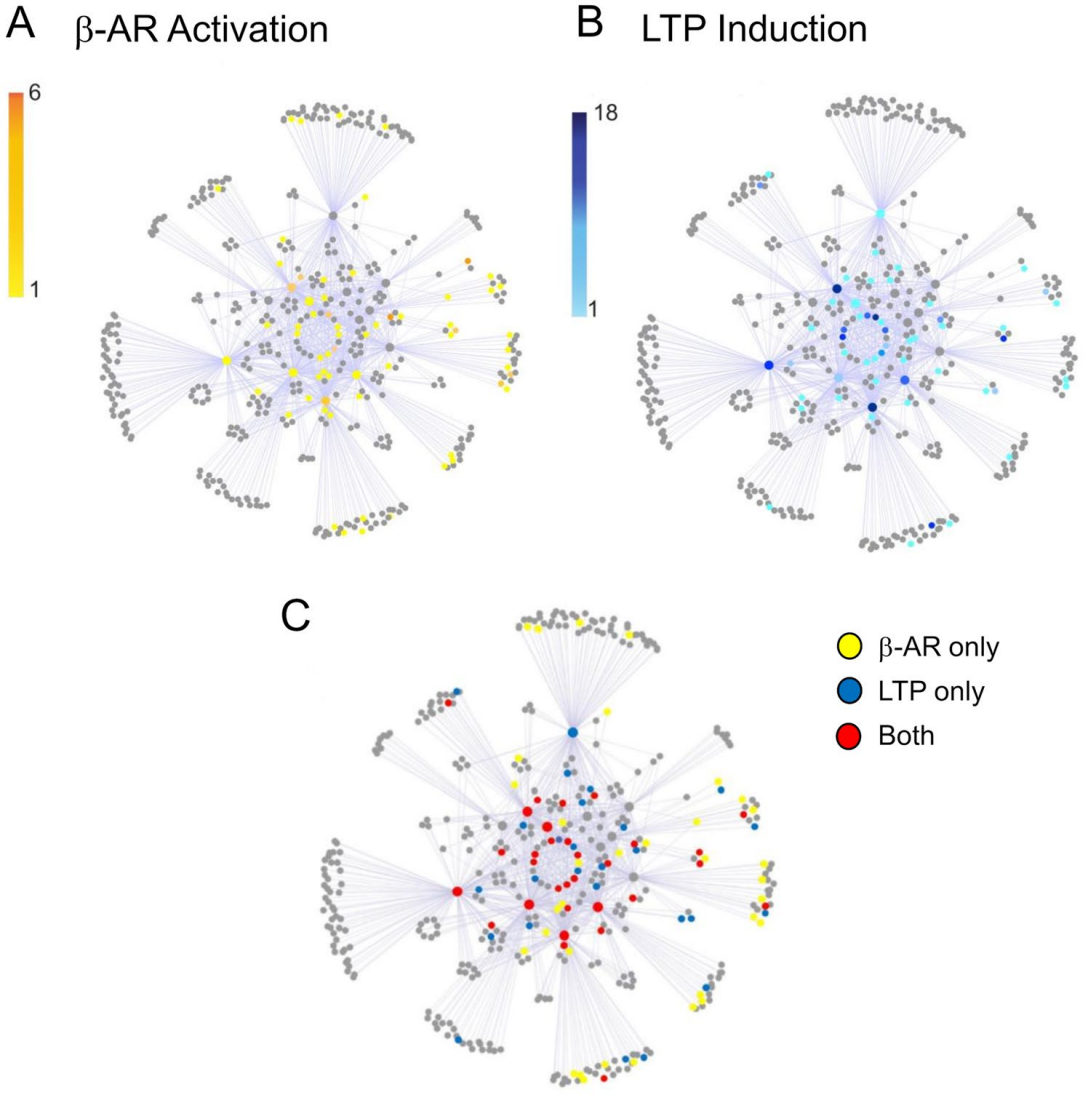
Increases in protein phosphorylation within the PSD protein interaction network induced by β-AR activation (**A**) and LTP induction (**B**). Results shown in (**B**) are from Li et al.^39^. Scale bars indicate number of regulated phosphorylation sites per protein. The number of phosphorylation sites regulated by β-AR per individual protein was lower than that seen following LTP induction (note difference in scalebars). The PSD protein interaction network was constructed using interactomes of different components of the PSD including Dlg4, Dlgap1, Shank3, Homer1, Syngap1, Agap2, Tsc1, Kalirin, Cnksr2, Tnik, Fmr1, Cyfip1, Cyfip2, Mycbp2, and Nckap1. (**C**) Sites within the PSD PIN regulated by only β-AR activation, only following the induction of LTP, or co-regulated by both activation of β-ARs and LTP induction.

Previously, we found that the induction of LTP by high-frequency synaptic stimulation increases protein phosphorylation preferentially on highly connected components within the PSD protein interaction network (PIN)^41^. This increase in phosphorylation was mainly due to the CAMK and AGC families of protein kinases, in particular CaMKIIα/β as well as PKC, PKA, and AKT1^41^. However, except for PKA, these protein kinases are not activated following β-AR activation. Indeed, β-AR activation induced changes in phosphorylation associated with decreased activity in multiple PKC isoforms as well as AKT1 (Supplementary Table 7). In addition, we found no evidence of CamKIIα/β activation using both MS and western immunoblotting (Supplementary Tables 1, 7). Thus, to gain insight into how ISO-induced changes in protein phosphorylation might facilitate LTP induction, we mapped how β-AR activation regulates protein phosphorylation across the PSD PIN. We built a PSD PIN using the interactomes of 15 target nodes including core scaffolds, kinases, cytoskeletal proteins, GTPases, Guanine exchange factors, and adaptors: Dlg4, Dlgap1, Shank3, Homer1, Syngap1, Agap2, Tsc1, Kalirin, Cnksr2, Tnik, Fmr1, Cyfip1, Cyfip2, Mycbp2, and Nckap1. This PSD PIN contained 1031 protein/protein interactions and we identified 303 regulated nodes within the network. Surprisingly, similar to what occurs following LTP induction^41^, we observed that β-AR activation leads to a preferential phosphorylation of highly connected nodes within the PSD PIN (Fig. 6A, B). Moreover, proteins that were phosphorylated by both β-AR activation and LTP induction were also preferentially located at highly connected nodes within the network (Fig. 6C). This suggests that highly connected nodes might share common phosphorylation sites regulated by both activation of NMDARs during LTP induction and β-AR activation. Consistent with this, we identified 10 phosphorylation sites in core scaffold components that were upregulated by both β-AR activation and LTP induction (Supplementary Table 9). Interestingly a number of these sites have been previously found by us and others to be phosphorylated by CaMKIIα^41,51,52^. However, ISO had no effect on CaMKIIα phosphorylation at its autophosphorylation site (T286), indicating CaMKIIα activity is not altered by β-AR activation. This suggests that PKA activation might be replacing CaMKII as a kinase for core components of the PSD scaffold machinery in β-AR-dependent forms of LTP.

## Discussion

Our results indicate that the induction of LTP by a diverse set of induction protocols, ranging in frequency from 5 to 100 Hz and in duration from a few seconds to 3 min, is highly β-AR dependent at SC fiber synapses onto CA1 pyramidal cells in the ventral hippocampus. Although HFS alone did induce some LTP at ventral SC fiber synapses, the amount of potentiation was smaller compared to that induced by HFS of SC fiber synapses in dorsal hippocampus (Fig. 2C, D). Notably, the relatively modest potentiation of ventral hippocampus SC fiber synapses produced by HFS is strongly facilitated by β-AR activation (Fig. 2D). Moreover, HFS-induced LTP at SC fiber synapses in the ventral, but not dorsal, hippocampal CA1 region is inhibited by β-AR antagonist propranolol^36^, suggesting that release of endogenous NE supports HFS-induced LTP at ventral SC synapses. Thus, NE release and activation of β-ARs appears to be an essential factor that, along with coincident pre- and postsynaptic activity, is required for LTP induction at SC fiber synapses onto CA1 pyramidal cells in the ventral hippocampus. In contrast, β-AR facilitates LTP induction in a more conventional, modulatory fashion at SC fiber synapses in the dorsal hippocampus^22–24,36^.

Although activation of SK-type potassium channels suppresses TPS-induced in the ventral hippocampus^37^, we find that the facilitation of LTP induction by the SK channel blocker apamin is highly activity-dependent. Thus, in the presence of apamin LTP induction exhibits a pronounced, inverted-U shape dependence on TPS train duration (Fig. 3). This suggests that multiple, distinct regulatory processes and signaling mechanisms underlie the induction of LTP induction by different duration TPS trains^47^. Consistent with this notion, protein phosphatase inhibitors facilitate the induction of LTP by TPS trains lasting one or more minutes, but have no effect on the induction of LTP by short (15–30 s) trains of TPS at SC fiber synapses in the dorsal hippocampus^22^. Unlike apamin, ISO enables the induction of LTP by all three TPS protocols at SC fiber synapses in the ventral hippocampus. This, and our analysis of PSD protein phosphorylation, suggests that β-AR signaling at excitatory synapses in the ventral CA1 region involves multiple downstream pathways that are able to interact with and modulate the distinct mechanisms underlying the induction of LTP by different patterns of TPS.

Results from previous studies suggest that the facilitation of LTP induction by β-AR agonists involves phosphorylation-dependent modulation of NMDARs^53^, AMPARs^28,31,48^, L-type voltage-gated Ca^2+^ channels^54^, and features important roles for the PKA adaptor protein AKAP5^55^ as well as regulators of protein phosphatase signaling^22,27^. Consistent with these findings, we find that phosphorylation of all of these proteins is regulated by β-AR at excitatory synapses in the ventral CA1 region. However, the majority of phosphorylation sites in these proteins identified in our experiments do not correspond to sites previously implicated in the modulation of LTP induction by β-AR activation. For example, in addition to increasing phosphorylation of AMPAR GluA1 subunits at S845, a site thought to have a crucial role in the β-AR modulation of LTP induction^28,31,48^, β-AR activation also increased phosphorylation at sites in the cytoplasmic C-terminal tails of AMPAR GluA2 and GluA3 subunits (S901 and S885, respectively). Interestingly, quantitative biochemical measurements of the AMPAR subunit phosphorylation have found that basal levels of GluA1 subunit phosphorylation at S845 in the hippocampus are vanishingly low, raising doubts about the role of GluA1 S845 phosphorylation in synaptic plasticity^56,57^ (however, see^58^). Moreover, the potentiation of excitatory postsynaptic currents induced by β-AR activation in hippocampal pyramidal cells is disrupted in GluA3, but not GluA1, null mutant mice^59^. Thus, future experiments investigating the functional effects of the GluA2/3 subunit phosphorylation at the sites identified in our experiments may help clarify the role of AMPAR phosphorylation in β-AR-dependent forms of LTP. Although a β-AR-mediated increase in phosphorylation of L-type (Cav1.2) calcium channels at S1928 is thought to have a crucial role in theta-frequency stimulation-induced LTP^54^, our MS analysis did not detect this site. β-AR activation did, however, increase phosphorylation at numerous sites in L-type calcium channel β2 and β4 subunits. Moreover, β-AR activation induced extensive changes in phosphorylation of R-type (Cav2.3) and P/Q-type (Cav2.1) voltage-dependent calcium channels. Indeed, amongst all the ligand-gated and voltage-activated channels regulated by β-AR activation, voltage-activated calcium channels exhibited the most pronounced changes in phosphorylation. Thus, our findings are not only consistent with the notion at L-type calcium channels have an important role in β-AR-dependent forms of LTP^54^ but also suggest that modulation of other types of calcium channels may be important as well.

Notably, we identified a number of novel phosphorylation sites regulated by β-AR activation. For example, although we did not detect phosphorylation of SK channels, our MS analysis did identify multiple phosphorylation sites in large-conductance BK-type potassium channels. Interestingly, Ca^2+^ influx via NMDARs^60,61^ and/or N-type voltage-dependent Ca^2+^ channels^62,63^ activates BK channels in neurons. Thus, during theta-frequency trains of synaptic activity, BK channel activation may oppose the strong dendritic spine depolarization needed to relieve the voltage-dependent Mg^2+^ block of NMDAR ion channels and induce LTP. Although the functional effects of the β-AR-regulated phosphorylation sites we identified in BK channels are unknown, the potential role of these channels in β-AR-dependent forms of LTP is an interesting question for future experiments. The bi-directional changes in phosphorylation induced at multiple sites in SynGAP1 and Lrrc7 (Densin-180) following β-AR activation are also intriguing. SynGAP1, a synaptic Ras GTPase activating protein, has a crucial role in synaptic plasticity^64,65^, as does the PSD scaffolding and CamKII binding protein Densin-180^66^. Interestingly, β-AR activation increased SynGAP1 phosphorylation at S1105. Phosphorylation of this site by CamKII during the induction of LTP is thought to trigger the translocation of SynGAP out of synapses, thereby enabling activation of small G proteins that have a crucial role in promoting changes in AMPAR trafficking and dendritic spine structure required for LTP^51^. β-AR activation also regulated phosphorylation of several proteins involved in small G protein signaling pathways responsible for plasticity of dendritic spine structure^67–69^, such as the NMDAR-associated Rho GTPase Arhgap32 (p250GAP)^70,71^ and the protein kinase PAK1^67,72^. Our identification of these, and other, novel targets of β-AR signaling suggest that the mechanisms underlying the facilitation of LTP induction by β-AR activation are surprisingly diverse and regulate molecular processes involved in the plasticity of both dendritic spine structure and function. Moreover, these results are in line with the diversity of molecular and cellular functions thought to be involved in the induction and maintenance of LTP.

Previous studies investigating the mechanisms underlying β-AR modulation of LTP induction have largely focused on examining the role of individual downstream targets modulated by PKA activation. Indeed, this was the approach we initially used to test the hypothesis that β-AR activation enables LTP induction by inhibiting SK-type K^+^ channels. This “single target”, PKA signaling-centric approach has merit, as it has provided several important insights into underlying mechanisms. Our MS analysis indicates, however, that β-AR activation regulates a large number of phosphorylation sites in PSD proteins with diverse functional and structural roles at synapses. Thus, rather than acting primarily via modulation of a single downstream target, it seems likely that changes in phosphorylation of multiple synaptic proteins collectively contribute to the ability of β-AR activation to enable LTP induction at SC fiber synapses onto ventral CA1 pyramidal cells. Moreover, β-AR activation not only leads to activation of PKA but also triggers activation of multiple PSD protein kinases, including PAK1, ERK1/2 and JNK3. β-AR activation also decreased phosphorylation sites that regulate SIK3, GSK3α, PKCα/β and PKG protein kinase activity. Thus, bidirectional regulation of multiple protein kinases may importantly contribute to the facilitation of LTP induction by β-AR activation. Notably, β-AR activation did not increase CamKIIα protein kinase activity in the ventral hippocampus. Thus, phosphorylation of AGC-type kinase sites likely primarily relies on PKA activation. Our results thus suggest that activation of β-ARs in the ventral hippocampus uses a distinct set of protein kinases to regulate core components of the PSD PIN. A number of these sites are localized within the core signaling machinery of the PSD PIN and many of them have been described to be essential for the induction of LTP. Therefore, the ability of β-AR activation to partially recapitulate changes in phosphorylation in the PSD PIN that occur during the induction of in LTP^41^ might importantly contribute to the crucial role of these receptors in the induction of β-AR-dependent forms of LTP. Notably, prenatal and juvenile stress induce long-lasting alterations in LTP induction and its modulation by β-AR activation in both the dorsal and ventral hippocampus^73,74^. In addition, dysregulation of noradrenergic signaling has been described in a number of psychiatric and neurodegenerative disorders, such as schizophrenia^75^, ADHD^76^ and Alzheimer’s disease^77^. Thus, the novel pathways and targets described here may also represent potential therapeutic targets for treating these disorders.

## Materials and methods

### Animals

Hippocampal slices were obtained from male, C57Bl/6N mice (Charles River Laboratories, 2–3 months old). Littermates were housed with no more than four mice/cage in a 12/12 light/dark cycle with food and water available ad libitum. Experiments and procedures were done in accordance with guidelines and regulations in the U.S. Public Health Service’s Policy on Humane Care and Use of Laboratory Animals and were approved by the Institutional Animal Care and Use Committee at the University of California, Los Angeles. Methods and experimental results are reported in accordance with ARRIVE guidelines.

### Slice preparation and electrophysiological recordings

Mice were deeply anesthetized with isoflurane and, following cervical dislocation, the brain was removed and rapidly placed in cold (~ 4 °C), oxygenated (95% O2/5% CO2) artificial cerebrospinal fluid (ACSF) containing 124 mM NaCl, 4 mM KCl, 25 mM NaHCO_3_, 1 mM NaH_2_PO_4_, 2 mM CaCl_2_, 1.2 mM MgSO_4_, and 10 mM glucose (all from Sigma-Aldrich). After allowing 4 to 5 min for the brain to cool, hippocampi from both hemispheres were dissected from the rest of the brain and 400-µm-thick slices were cut using a manual tissue slicer. Slices were then transferred to an interface-type chamber perfused (2–3 ml/min) with warm (30 °C) ACSF and allowed to recover for a least 2 h. Slices obtained from the ventral third of the hippocampus were used in all experiments except for those shown in Figs. 1A and 2D, where experiments were done using slices from the dorsal third of the hippocampus. A glass microelectrode filled with ACSF (~ 10 MΩ resistance) was placed in stratum radiatum of the CA1 region to record field excitatory postsynaptic potentials (fEPSPs) elicited by SC/commissural fiber stimulation. Stimulation was delivered using a bipolar stimulating electrode placed in stratum radiatum (0.02 ms duration pulses, basal stimulation rate = 0.02 Hz). The maximal fEPSP amplitude that could be evoked was determined at the start of each experiment and the intensity of presynaptic fiber stimulation was set to elicit fEPSPs with amplitudes that were approximately 50% of the maximal response. To determine the role of β-AR activation in synaptic plasticity we examined the effects of different duration trains of theta-pulse stimulation (TPS) delivered alone or following a 10-min bath application of the β-AR agonist ISO (1.0 µM). Unlike the more commonly used theta-burst stimulation protocols^42^, TPS trains consisted of single pulses of presynaptic fiber stimulation delivered at 5 Hz. In some experiments we also induced LTP using a high-frequency stimulation (HFS) protocol consisting of two, one-second-long trains of 100 Hz stimulation delivered with an inter-train interval of 10 s. The average slope of fEPSPs recorded 40–45 min post-TPS or 55–60 min post-HFS (normalized to baseline) were used for statistical comparisons. Statistical significance for results from electrophysiological experiments were analyzed using Student t-tests or, where appropriate, one-way or two-way ANOVAs with Bonferroni post hoc comparisons. Statistical tests were performed using SigmaPlot 12.5 (Systat Software, Inc.) or Microsoft Excel. Results are reported as mean ± SEM and full results from statistical tests are provided in the figure legends.

### Biochemical and MS analysis of PSD protein phosphorylation

Slices from the ventral hippocampus were prepared as described above with the exception that following slicing the dentate gyrus, CA3 region, and the subiculum were removed to produce “mini-slices” containing just the CA1 region. CA1 mini-slices were then transferred to interface-type chambers perfused with ACSF. After allowing the slices to recover for 2 h, half of the mini-slices from each hippocampus were snap-frozen by transferring them into a pre-frozen microcentrifuge tube placed on a bed of crushed dry ice to serve as untreated control tissue. The remaining slices were collected in the same manner after a 10-min bath application of ACSF containing 1.0 µM ISO. For each experiment (n = 3), we pooled 10 CA1 mini-slices (obtained from the ventral hippocampi of three mice) for each condition. Using techniques described elsewhere^41^, we prepared isolated PSD fractions from these samples and, following enrichment of phosphopeptides with titanium dioxide (TiO2) chromatography, samples were analyzed using LC–MS/MS. Samples reconstituted in LC buffer A (0.1% formic acid in water), randomized, and then injected onto an EASY-nLC 1200 ultra-high-performance liquid chromatography coupled to a Q Exactive Plus quadrupole-Orbitrap mass spectrometer (Thermo Fisher Scientific). Peptides were separated by a reverse phase analytical column (PepMap RSLC C18, 2 µm, 100 Å, 75 µm × 25 cm). Flow rate was set to 300 nL/min at a gradient from 3% LC buffer B (0.1% formic acid, 80% acetonitrile) to 38% LC buffer B in 110 min, followed by a 10-min washing step to 85% LC buffer B. The maximum pressure was set to 1,180 bar, and column temperature was maintained at 50 °C. Peptides separated by the column were ionized at 2.4 kV in positive ion mode. MS1 survey scans were acquired at the resolution of 70,000 from 350 to 1800 m/z, with a maximum injection time of 100 ms and AGC target of 1e6. MS/MS fragmentation of the 14 most abundant ions were analyzed at a resolution of 17,500, AGC target 5e4, maximum injection time 65 ms, and normalized collision energy of 26. Dynamic exclusion was set to 30 s, and ions with charge + 1, + 7 and > + 7 were excluded.

MS/MS fragmentation spectra were searched with Proteome Discoverer SEQUEST (version 2.2, Thermo Scientific) against in silico tryptic digested Uniprot all-reviewed Homo sapiens database (release June 2017, 42,140 entries). The maximum missed cleavages was set to two. Dynamic modifications were set to phosphorylation on serine, threonine, or tyrosine (+ 79.966 Da), oxidation on methionine (+ 15.995 Da), and acetylation on protein N-terminus (+ 42.011 Da). Carbamidomethylation on cysteine (+ 57.021 Da) was set as a fixed modification. The maximum parental mass error was set to 10 ppm, and the MS/MS mass tolerance was set to 0.02 Da. The false discovery threshold was set strictly to 0.01 using the Percolator Node. Individual phospho-site localization probabilities were determined by the ptmRS node, and phospho-sites with < 0.75 localization probability were removed. The relative abundance of phospho-peptides was calculated by integration of the area under the curve of the MS1 peaks using the Minora LFQ node in Proteome Discoverer. No data imputation was performed for missing values. Phospho-peptides were filtered so that each condition had at least two quantified values. Phospho-peptide intensities were then normalized by log2-transformation and sample median subtraction.

## Supplementary Information

Supplementary Tables.

Supplementary Table 10.

## Data Availability

The dataset for the LC–MS/MS experiments is provided in Supplementary Table 1 and the dataset for all electrophysiological experiments is provided in Supplementary Table 10.
